# The Task‐Sharing Path to Safe and Accessible Anaesthesia Care in India: The Role of Professional Associations in Health Policy Reform

**DOI:** 10.1002/puh2.205

**Published:** 2024-06-24

**Authors:** Nobhojit Roy, Pranav Bhushan, Monali Mohan, Amal Paonaskar

**Affiliations:** ^1^ Department of Global Public Health Karolinska Institutet Stockholm Sweden; ^2^ The George Institute for Global Health New Delhi India; ^3^ Ministry of Health and Family Welfare Government of India New Delhi India; ^4^ Dept of Surgery Indraprastha Apollo Hospital New Delhi India; ^5^ World Federation of Societies of Anaesthesiologists (WFSA) London UK

**Keywords:** anaesthesia capacity, global surgery, health policy, India, national surgical plans

## Abstract

The worldwide anaesthesia workforce shortage is a concern and ‘shared responsibility’ for all the national or state anaesthetic societies and Ministries of Health. The Lancet Commission on Global Surgery estimated the need for 143 million additional surgeries each year globally. These would be included in the World Health Organization's (WHO) 44 essential surgeries to be performed at district hospitals. However, insufficient availability of safe anaesthesia is a key barrier. The World Federation of Societies of Anaesthesiologists (WFSA) recommends at least five specialist physician‐anaesthesia providers per 100,000 population. India requires at least 60,000 additional physician‐anaesthesiologists over the next 10 years. This paper discusses the two‐decade journey of a policy initiative by the Ministry of Health and Family Welfare (MoHFW) in India to create a new category of a physician with Life Saving Anaesthetic Skills (LSAS). This addressed the shortage of anaesthetists specifically for the dire emergency obstetric situations at the First Referral hospitals. The annual training capacity for physicians for LSAS training was 40–100 in 5 of the poorest states of India, with the maximum shortages of anaesthetists. On following up a sample of 838 LSAS physicians, only about two‐thirds were able to practice their life‐saving skills. The MoHFW innovated further by pairing a physician trained in Emergency Obstetric Care with an LSAS‐physician (buddy‐pairing) as a functioning team. For comparison, we discuss the midwife model supported by the professional association of obstetricians in India. The flexible, ‘team‐based’ task‐sharing approach optimizes anaesthesia care within available resources. Leadership and vision from the professional societies of anaesthesiology are key to policy reform in India. National engagement can be facilitated through support for district‐level non‐specialist physician provider with life‐saving anaesthesia skills training, engagement in research and formulation of the national surgical and anaesthesia plans to achieve universal healthcare in India.

## 1 Introduction

The worldwide anaesthesia workforce shortage is a concern and ‘shared responsibility’ for all the national or state anaesthetic societies and Ministries of Health [1]. This policy review addresses the shortage of anaesthesiologists in India and underscores the pivotal role of champions within the professional societies of anaesthesiologists to address this national unmet need [1]. The recent COVID‐19 pandemic has highlighted the stark and unmet need of essential critical care and safe anaesthesia and acute global shortage of anaesthetists [2].

## 2 The Unmet Need in Surgery and Workforce Shortage

The Lancet Commission on Global Surgery (LCoGS) estimates the need for 143 million additional surgeries each year globally. Most are part of the World Health Organization's (WHO) 44 essential surgeries, which are expected to be performed at district hospitals. The Bellwether procedures of caesarean section, surgery for peritonitis and compound fracture are the most important life‐saving surgeries on this unmet needs list [3]. Insufficient availability of safe anaesthesia is a key barrier to performing these surgeries [4].

The LCoGS goal for the global surgical workforce (surgeons, anaesthetists, obstetricians = SAO) is to achieve at least 20 SAOs per 100,000 population by 2030 [3]. As per global observational studies, lower SAO workforce density has been strongly correlated with higher patient mortality rates, such as maternal mortality [6]. The World Federation of Societies of Anaesthesiologists (WFSA) recommends at least 5 specialist physician‐anaesthesia providers (PAP) per 100,000 population. According to the global anaesthesia workforce survey, India is steady at 1.27 anaesthetists (PAP) per 100,000 [5, 6]. Therefore, India needs to quadruple its workforce to achieve the minimum standards set by the LCoGS and WFSA over the next 10 years [1]. However, training 60,000 physician‐anaesthesiologists in 10 years is a near‐impossible task.

Countries that have achieved the ideal anaesthesiologist‐to‐population ratio have adopted the model of the anaesthesia care team, in which anaesthesia tasks are shared by qualified team members and typically overseen by a physician who has specialized in anaesthesia [6, 7]. High‐income countries (HICs) routinely employ providers other than physician specialists, who have competencies to perform specific tasks, with medical cover provided by their training. Canada has Anaesthetic Assistants, whereas the United Kingdom has the ‘four hands’ model, which includes a technician called the Operating Department Practitioner. In Switzerland, an anaesthesia team has three people: the physician‐specialist, nurse anaesthetist and anaesthetist assistant. Sweden has more non‐physician providers of anaesthesia than physician providers [7]. However, some low‐and‐middle‐income countries, such as India, that suffer from a huge shortage of anaesthetists only allow physician‐anaesthesia specialists to practice and perform the full scope of anaesthesia tasks from the simplest to most complex [8].

The WFSA has defined categories of providers in their registry: specialist physician provider (physician with a formal anaesthetic qualification), a non‐specialist physician provider (physician without formal anaesthetic training), nurse provider (nurse with either formal or informal anaesthetic training) and non‐physician/non‐nurse provider (other health worker with either formal or informal anaesthetic training) [6]. India has no recognized non‐PAP (NPAP). The Indian Society of Anaesthesiologists (ISA), a member of the WFSA, has confirmed that only physician‐anaesthesiologists will cater to all the anaesthesia needs of India and supports no other categories of anaesthesia providers [5].

Unfortunately, the reality of India is quite different. NPAP remains the main provider of anaesthesia care for most life‐saving surgeries at the district level and in rural areas, where there are no physician‐anaesthetists. Therefore, someone must step in to fill the void of primary anaesthesia provision for essential life‐saving surgeries. In some cases, the *dais* (traditional midwife) in obstetrics, OT *babus* (technicians), nurses and surgeons themselves fill the void of the missing anaesthetists. Furthermore, owing to the limited availability of spinal anaesthesia, Ketamine and open Ether are the main anaesthetic agents used by less‐experienced anaesthesia providers for essential and life‐saving surgery in 2020 [9]. Even if the impossible gap of 60,000 physician‐anaesthesiologists (who have the longest training time) is filled, few will ever practice in rural areas. An excess of anaesthesiologists will further skew the urban–rural distribution, super‐saturate urban health facilities and further reduce their professional fees [8].

Ironically, HICs, such as the USA, Sweden and Australia, who have achieved the recommended anaesthetist‐to‐population ratio, have included NPAP or General Practitioner Anaesthetists in their workforce, despite their litigious work environment. Key opinion leaders in anaesthesiology realized that ‘task‐sharing’ was the only way to achieve an adequate and well‐utilized anaesthesia workforce [1, 8]. These highly regulated countries share anaesthetic tasks with other non‐physician providers and ensure that they are performed at a safety standard equivalent to that by a physician.

This paper discusses the two‐decade journey of a policy initiative by the Ministry of Health in India to create a new category of a physician healthcare worker with Life Saving Anaesthetic Skills (LSAS) specifically for Obstetric emergencies. These flexible, ‘team‐based’ approaches optimize anaesthesia care within the available resources and are not novel models. They have been successfully adopted by many disciplines; we discuss obstetrics as a speciality in India as a comparison.

## 3 Example of Federation of Obstetric and Gynaecological Societies of India (FOGSI) Leadership

The FOGSI has been in the forefront of addressing the shortage of obstetricians in India [10]. In the 1960s and 1970s, the FOGSI supported the extensive *Dai*‐training programs in safe and basic obstetric practices across many states. The traditional birth attendants (*Dais*) were not physicians and only had a school education, if at all; therefore, they were not legitimate agents of science. Despite this, FOGSI recognized and co‐opted them as their extensions for areas with high homebirths in states that were unable to provide doctors. The FOGSI has been internationally applauded for ushering in a revolution for safer births in India.

A major learning was that a protocolized approach for primary and essential obstetrics, implemented by available non‐specialist health workers, saved more lives and was more sustainable and appropriate for the low‐resource context [8, 11]. Learning from Thailand, Nigeria and the USA, the Steering Committee on Family Welfare for the Tenth Five Year Plan (2002–2007) recommended that the shortfall of obstetricians and gynaecologist specialists at the district level should be filled by upskilling existing manpower, while training specialist obstetricians [12].

Working alongside the Ministry of Health and Family Welfare (MoHFW), the FOGSI, as a technical advisor, responded by starting a short course for physicians in Emergency Obstetrics (EmOC). EmOC training for C‐sections has remarkably mitigated the shortages of obstetricians at the district level over the last two decades, with emergency obstetricians sharing the on‐call rota and reducing specialists’ emergency load. By co‐opting dais and EmOCs through task‐sharing, the FOGSI led from a position of strength and respect and freed up time and bandwidth for more complex gynaecological elective and innovative obstetric work [10].

Conversely, the ISA had a different approach. The steering committee had observed that ‘shortage of anaesthetists’ was the single most important cause of inadequacy of emergency care in government hospitals, particularly in rural areas. Subsequently, the ISA filed a public interest litigation (PIL) against the MoHFW‐led short course called the LSAS for Emergency Obstetric Care [13]. In 2002, the MoHFW, the WHO and European Commission (EC) had initiated this short‐course training curriculum for MBBS Doctors, to issue an LSAS competency certification. The Delhi High Court quashed the PIL and strongly stated that the course was formulated in ‘national interest’. The judgement stated that the ISA should not feel ‘insecure’, and the LSAS was not an attempt to replace specialist anaesthesiologists. It stated ‘LSAS is not an additional qualification/specialization to practice anaesthesiology, but only to tackle the emergency obstetric situations at First Referral Unit (FRU) level’ [13]. The MoHFW further innovated the LSAS approach by incentivizing pairing an EmOC‐trained physician with an LSAS‐physician (buddy‐pairing) as functioning teams within the district‐level FRUs to mitigate the manpower shortage [7, 8].

Although the ISA had to be reminded of its national duty, the FOGSI led with professional pride and marched ahead in the same year and transformed maternal healthcare in India, with their population stabilization and rural women's health program (2003), technological advances for the underprivileged (2004), optimization of labour management to reduce maternal mortality (2005) and youth health (2006). More recently, under the Pradhan Mantri Surakshit Matritva Abhiyan (PMSMA), the Govt. of India‐FOGSI joint corporate social responsibility initiative, a response to the Prime Minister's call to all private practitioners, provides a fixed day assured, comprehensive and quality antenatal care to pregnant women on the ninth of every month [14]. Similarly, Manyata, a FOGSI‐led quality improvement and assurance program, is well‐aligned with the latest MoHFW initiative called Laqshya. Laqshya ensures the best clinical practices in the labour room and operation theatres by well‐trained and sensitized staff to provide a better, respectful and safer experience for women during childbirth. The FOGSI is a core member of all national‐level standard treatment guidelines (STGs) developed by the MoHFW [10].

## 4 ISA's Leadership Role in Safe Anaesthesia

The global movement towards safe anaesthesia and surgery is an opportunity for the ISA to be leading the movement in India as expert technical partners to the MoHFW's *Laqshya* program for safer operation theatres and use of the surgical safety checklist [15]. The WFSA‐led Safer Anaesthesia from Education (SAFE) courses, which enhance recovery after surgery programs, and WFSA‐suggested initiatives are steps in this direction [1]. However, most surgical safety initiatives currently remain restricted to elite hospitals and medical colleges in metropolitan India. The Association of Rural Surgeons of India (ARSI) [16] has done valuable service to the cause of rural surgical access, but there is no Rural Anaesthesiologist association to share this responsibility.

Although support for NPAP (analogous to *Dai*‐support) is a pipedream, the ISA can begin supporting their non‐specialist physician‐colleagues who provide essential and primary anaesthesia care in rural India and make practices safer. The MoHFW‐led LSAS‐course is best placed for support and supervision [8, 11, 17, 18]. The course has been upgraded from a 16‐ to 24‐week training program, with a stronger skill‐based and latest evidence‐based curriculum. The course includes didactic lectures, mannequin‐based simulation training, 100 cases of spinal anaesthesia, 30 of general anaesthesia and 10 of epidural anaesthesia and laryngeal mask use.

Only two‐thirds of those who complete their LSAS training are able to practice life‐saving anaesthesia [11]. Hence, building the confidence of LSAS physicians, who are largely satisfied with their short multiskilling course, is a requirement. However, support through the initial period of post‐training and periodic refresher workshops would also be useful [11, 17].

Nonetheless, further mentoring, periodic evaluation and peer‐support are required when posted at the FRUs, a need catered to by the state health departments and development agencies. The ISA can professionally contribute here. Similar to the USA, specialist‐anaesthetists in India will be able to manage additional operative cases with increased remuneration and free time, by task‐shifting some simpler yet essential competencies to a functioning LSAS cadre and other anaesthesia providers. Table 1 represents collated data from State Health System Resource centres [19] and demonstrates that the LSAS program has a two‐decade history of rendering service to the populations most prone to ‘catastrophic’ medical expenditure through surgery. Healthcare expenses are the single most important cause of poverty creation in India, pushing landed labourers to sell their farmland to pay for medical bills, thus becoming impoverished landless ones [3]. Though acknowledged by the MoHFW, in the eyes of the ISA, LSAS‐trained physicians are considered to be no better than non‐PAPs in India, despite their remarkable contribution to Indian healthcare [8, 11].

**TABLE 1:** A snapshot of the critical gap‐filling by physicians with life‐saving‐anaesthesia‐skills (LSAS) over the last two decades in five poorer states of India of Odisha, Uttarakhand (UK), Bihar, Madhya Pradesh (MP), Maharashtra (MH) and Jharkhand (JH).

State	Odisha	UK	Bihar	MP	MH	JH
Total no. of LSAS‐trained doctors in the state	170	18	202	90	194	164
No. of LSAS‐trained doctors posted in the First Referral Unit hospitals	90	12	98	24	93	24
No. posted in other primary healthcare centres	15	0	60	21	57	87
Not in service for various reasons^a^	65	6	44	45	44	2

^a^’Not in service’ implies that LSAS‐trained MOs are in administrative positions, non‐clinical positions, on study leave, in other programs as program officers, resigned, suspended, on leave without permission, absconded or dead. Since 2006, the annual capacity ranges from 40 to 100.

Like FOGSI, the ARSI, Indian Association of Paediatric Surgeons and rural chapter of the Association of Surgeons of India (ASI) have greatly contributed toward improving access to surgery in India. However, similar interest groups for safe anaesthesia in the low‐resourced environment among Indian anaesthesia associations are missing. Individual anaesthesiologists have led movements of national interest in India in domains, such as Palliative Care; however, as a specialist association, a larger national vision and shared responsibility is warranted [20]. Senior Indian opinion leaders in anaesthesiology and the board of the ISA are key to changes in India. Engagement with service delivery, establishing appropriate regulatory frameworks and enhancing ethical practice guidelines can mitigate and distract from the insecurity, burn‐out and negativism of the dependent anaesthesia practice in India [21]. National engagement can be facilitated through support to our LSAS peers, engaging in research, formulating Indian STGs and contributing towards a robust health policy to achieve universal healthcare in India. In the future, maybe the ISA will engage with the policy‐makers, health authorities, the surgical and obstetric societies to meet the needs of the district hospitals and FRUs and lead the development of a framework and training for non‐PAPs.

## 5 Conclusion

Leadership and vision from the professional societies of anaesthesiology are key to policy reform in India. National engagement can be facilitated through support for district‐level non‐specialist physician providers with life‐saving anaesthesia skills training, engagement in research and formulation of national surgical and anaesthesia plans to achieve universal healthcare in India.

## Author Contributions

**Nobhojit Roy**: conceptualization, methodology, writing–review and editing, writing–original draft, data curation, supervision, project administration, validation. **Pranav Bhushan**: methodology, data curation, validation, writing–review and editing, writing–original draft, conceptualization. **Monali Mohan**: validation, writing–review and editing, methodology. **Amal Paonaskar**: data curation, validation, writing–review and editing.

## Conflicts of Interest

The authors declare no conflicts of interest.

## Data Availability

All data used in this policy review is available in the public domain, at the websites of the Ministry of Health (https://www.mohfw.gov.in/, https://nhsrcindia.org/), India and in the following references.
